# High detection rate for disease-causing variants in a cohort of 30 Iranian pediatric steroid resistant nephrotic syndrome cases

**DOI:** 10.3389/fped.2022.974840

**Published:** 2022-09-22

**Authors:** Maryam Najafi, Korbinian M. Riedhammer, Aboulfazl Rad, Paria Najarzadeh Torbati, Riccardo Berutti, Isabel Schüle, Sophie Schroda, Thomas Meitinger, Jasmina Ćomić, Simin Sadeghi Bojd, Tayebeh Baranzehi, Azadeh Shojaei, Anoush Azarfar, Mahmood Reza Khazaei, Anna Köttgen, Rolf Backofen, Ehsan Ghayoor Karimiani, Julia Hoefele, Miriam Schmidts

**Affiliations:** ^1^Genome Research Division, Human Genetics Department, Radboud University Medical Center, Nijmegen, Netherlands; ^2^Pediatric Genetics Division, Center for Pediatrics and Adolescent Medicine, University Hospital Freiburg, Freiburg University Faculty of Medicine, Freiburg, Germany; ^3^Institute of Human Genetics, Klinikum rechts der Isar, Technical University of Munich, School of Medicine, Munich, Germany; ^4^Department of Nephrology, Klinikum rechts der Isar, Technical University of Munich, School of Medicine, Munich, Germany; ^5^Cellular and Molecular Research Center, Sabzevar University of Medical Sciences, Sabzevar, Iran; ^6^Department of Medical Genetics, Next Generation Genetic Polyclinic, Mashhad, Iran; ^7^Children and Adolescents Health Research Center, Research Institute of Cellular and Molecular Science in Infectious Diseases, Zahedan University of Medical Sciences, Zahedan, Iran; ^8^Department of Biology, University of Sistan and Baluchestan, Zahedan, Iran; ^9^Department of Medical Genetics and Molecular Biology, School of Medicine, Iran University of Medical Sciences, Tehran, Iran; ^10^Pediatric Nephrology, Kidney Transplantation Complications Research Center, Mashhad University of Medical Sciences, Mashhad, Iran; ^11^Department of Pediatrics, Faculty of Medicine, Mashhad Medical Sciences, Islamic Azad University, Mashhad, Iran; ^12^Institute of Genetic Epidemiology, Faculty of Medicine and Medical Center - University of Freiburg, Freiburg, Germany; ^13^Center for Integrative Biological Signaling Studies, University of Freiburg, Freiburg, Germany; ^14^Bioinformatics Group, Department of Computer Science, University of Freiburg, Freiburg, Germany; ^15^Next Generation Genetic Polyclinic, Mashhad, Iran; ^16^Genetics Research Centre, Molecular and Clinical Sciences Institute, St. George's University, London, United Kingdom

**Keywords:** nephrotic syndrome, Iran, SRNS, *ARHGDIA*, *NUP205*, *COQ6*, *SGPL1*

## Abstract

**Background:**

Steroid resistant nephrotic syndrome (SRNS) represents a significant renal disease burden in childhood and adolescence. In contrast to steroid sensitive nephrotic syndrome (SSNS), renal outcomes are significantly poorer in SRNS. Over the past decade, extensive genetic heterogeneity has become evident while disease-causing variants are still only identified in 30% of cases in previously reported studies with proportion and type of variants identified differing depending on the age of onset and ethnical background of probands. A genetic diagnosis however can have implications regarding clinical management, including kidney transplantation, extrarenal disease manifestations, and, in some cases, even causal therapy. Genetic diagnostics therefore play an important role for the clinical care of SRNS affected individuals.

**Methodology and results:**

Here, we performed *NPHS2* Sanger sequencing and subsequent exome sequencing in 30 consanguineous Iranian families with a child affected by SRNS with a mean age of onset of 16 months. We identified disease-causing variants and one variant of uncertain significance in 22 families (73%), including variants in *NPHS1* (30%), followed by *NPHS2* (20%), *WT1* (*7*%) as well as in *NUP205, COQ6, ARHGDIA, SGPL1*, and *NPHP1* in single cases. Eight of these variants have not previously been reported as disease-causing, including four *NPHS1* variants and one variant in *NPHS2, ARHGDIA, SGPL1*, and *NPHP1* each.

**Conclusion:**

In line with previous studies in non-Iranian subjects, we most frequently identified disease-causing variants in *NPHS1* and *NPHS2*. While Sanger sequencing of *NPHS2* can be considered as first diagnostic step in non-congenital cases, the genetic heterogeneity underlying SRNS renders next-generation sequencing based diagnostics as the most efficient genetic screening method. In accordance with the mainly autosomal recessive inheritance pattern, diagnostic yield can be significantly higher in consanguineous than in outbred populations.

## Introduction

Mammalian kidneys fulfill a tremendous job by filtrating enormous amounts of blood without loss of essential components such as proteins in the urine. The glomerular filtration barrier consisting of three major layers [podocyte foot processes and the slit diaphragm, the glomerular basement membrane (GBM), and glomerular endothelial cells (GEC)] hereby ensures that proteins larger than 60 kD remain within the blood vessels. Smaller filtrated proteins are subsequently largely re-absorbed within the tubular system. Failure of this fine-tuned system results in proteinuria. Loss of large amounts of protein in the urine can result in nephrotic syndrome, characterized by proteinuria of more than >3.5 g per 1.73 m^2^ body surface area per day, hypoproteinemia (serum albumin is reduced to <2.5 g/dl), hyperlipoproteinemia, and edema. Acute complications include thrombosis, pleural effusions and ascites. Over time, growth restriction in children as well as progressive loss of podocytes with subsequent decline in renal function become evident (1). Two main clinical entities of nephrotic syndrome are distinguished: steroid sensitive nephrotic syndrome (SSNS) and steroid resistant nephrotic syndrome (SRNS). Steroid resistant nephrotic syndrome is defined as persistent nephrotic-range proteinuria after 4 weeks of oral prednisone at 60 mg/m^2^ per day (2). Steroid resistant nephrotic syndrome is a rare condition, however represents one of the most common causes of childhood-onset kidney failure, occurring as frequently as 1:10,000 in some populations (3). Histopathological findings are most often focal segmental glomerulosclerosis (FSGS) (4). Disease-causing variants in over 60 genes have been described to date to cause monogenic forms of SRNS, explaining approximately one-third of all SRNS cases with an age of onset <25 years (5). Many disease-associated genes encode for proteins essential for proper podocyte function. Most commonly, disease-causing variants affecting the slit diaphragm proteins Nephrin (*NPHS1*) and Podocin (*NPHS2*) or the transcription factor WT1 or LAMB2 are identified (6). Hereditary SRNS usually does not respond to immunosuppressive treatment and, in contrast to SSNS, has a poor prognosis with regards to renal survival: 10 years after onset of proteinuria, SSNS patients experience end-stage kidney failure (ESKF) in <5% of cases while ESKF occurs in 50% of individuals with sporadic SRNS and 80% of genetically proven SRNS cases (4, 7). However, disease re-occurrence after kidney transplantation is less likely in most hereditary SRNS cases compared to sporadic SRNS cases (8). Identification of an underlying genetic cause in SRNS individuals therefore has direct therapeutic consequences. Childhood-onset SRNS occurs more frequently in consanguineous populations as it is often autosomal recessively inherited (9, 10).

In this study, we describe the genetic findings using exome sequencing (ES) in 29 Iranian children with SRNS from consanguineous parents with as well as one Iranian parent pair where only insufficient DNA of the affected child was available.

## Methods

### Study population

The clinical diagnosis of SRNS was defined as absence of complete remission after 4 weeks of daily prednisone therapy at a dose of 60 mg/m^2^ per day for all affected individuals. Informed consent for genetic diagnostics was obtained from all participants of this study or their legal guardians and genetic testing was conducted in accordance with the standards of the 2013 Helsinki declaration. This study was approved by the local ethics committees of Nijmegen, Netherlands, Freiburg und Munich, Germany and samples processed under the Radboudumc Diagnostics Innovation programme (CMO2006-048) to establish a genetic diagnosis underlying the patient's health disturbances.

### DNA extraction

Genomic DNA was extracted from whole blood, using standard salting out method. The concentration of DNA was measured by Qubit 2.0 (Life Technologies, Carlsbad, CA, USA).

### PCR and Sanger sequencing

Samples were first tested for the presence of previously reported recurrent (likely) disease-causing variants in *NPHS2* exons 5 and 7 (11) using PCR followed by Sanger sequencing. Likewise, segregation of variants identified was verified by PCR and Sanger sequencing in parents unless trio (i.e., case-parent) exome analysis had been performed. Conventional PCR was performed by Taq polymerase (Roche, Mannheim, Germany) based on manufacturer's instruction. Primer sequences are available upon request.

### Exome sequencing

Two to five micrograms of DNA were subjected to ES and exome capture was performed using Agilent SureSelect Human All Exon V6 Kit. Paired-end sequencing on a HiSeq 2500 Genome Analyzer (Illumina, San Diego, CA, USA) was performed and build hg19 of the UCSC Genome Browser was used as a reference genome. VarScan version 2.2.5, Mutect, and GATK Somatic Indel Detector were used to detect SNVs and indels, respectively. Filtering was performed as previously described (12). In brief, a minor allele frequency cutoff of 1% in gnomAD (gnomAD, http://gnomad.broadinstitute.org) was applied and the remaining variants were filtered first for genes in which SRNS disease-causing variants had been previously published (13). If no disease-causing variants could be identified using this strategy, BAM files were manually inspected for homozygous CNVs in these genes and exome data further analyzed for disease-causing variants in other genes not previously associated with SRNS. Variants rated as “likely pathogenic” or “pathogenic” as per ACMG criteria and current amendments (14) are summarized as “disease-causing” throughout the text.

## Results

Molecular genetic diagnostics were undertaken for 30 Iranian unrelated consanguineous families with at least one child clinically diagnosed with SRNS. In one family (sample 3), insufficient DNA of the affected child was available for genetic testing by ES, therefore both parents were tested by ES instead, followed by Sanger sequencing of the child.

**Figure 1:**
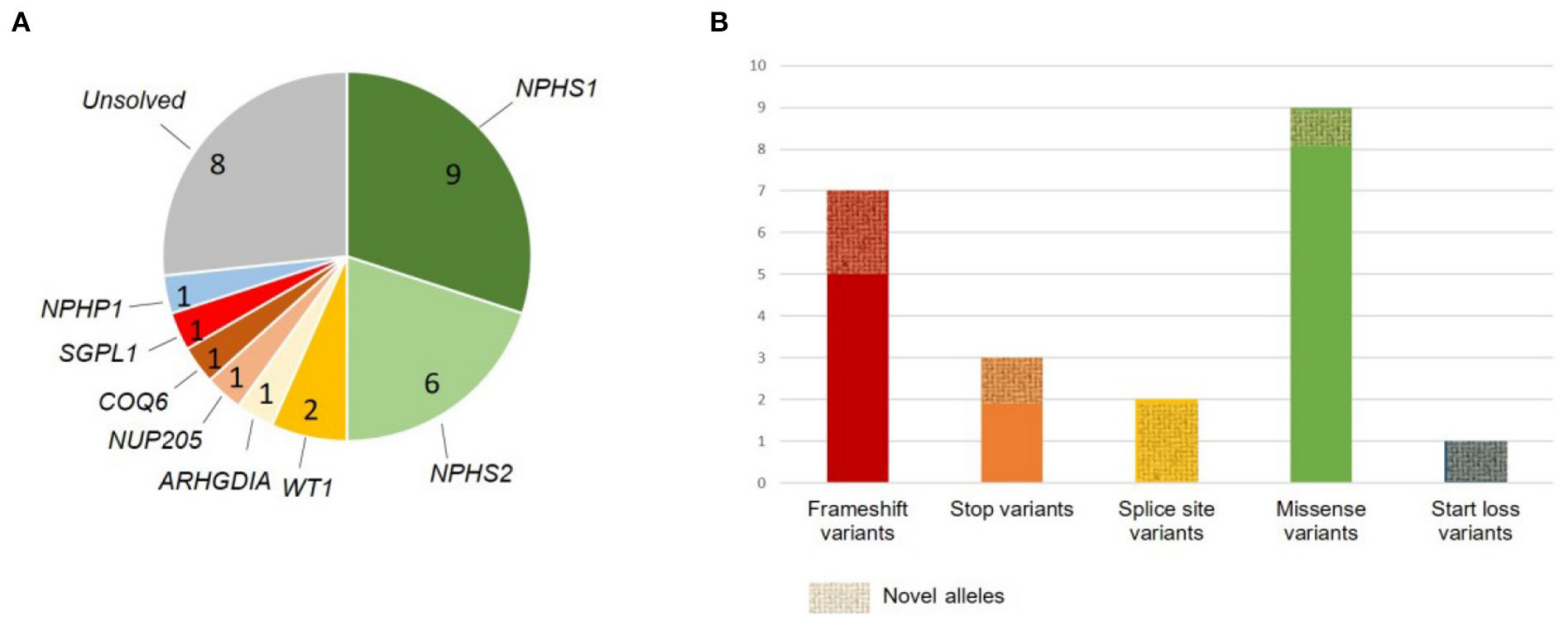
Summary of genetic findings. **(A)** Gene-specific distribution of identified disease-causing variants and one variant of uncertain significance; unsolved cases are shown in gray. **(B)** Variant type distribution. Alleles not previously reported are marked as novel alleles.

In total, we identified an underlying genetic cause in 21/30 families and a variant of uncertain significance (VUS) in one family (diagnostic yield: 73%) (Table 1). All identified disease-causing variants locate to genes known to cause monogenic SRNS when dysfunctional with the exception of one case carrying a variant in a nephronophthisis gene (*NPHP1*) instead. The gene most frequently affected was *NPHS1* (MIM# 602716; nine families: 38% of solved families and 30% of all families), followed by *NPHS2* (MIM# 604766; six families: 29% of solved families, 20% of all families). Two cases were found to be caused by *WT1* variants (MIM# 607102; 10% of solved families, 7% of all families), while variants in *NUP205* (MIM# 614352)*, COQ6* (MIM# 614647), *ARHGDIA* (MIM# 601925), and *SGPL1* (MIM# 603729) were identified in single families (5% of solved families, 3% of all families). In addition, we identified a disease-causing deletion in *NPHP1* [MIM# 607100; gene previously associated with nephronophthisis (NPHP)] in one family (5% of solved families, 3% of all families; Figure 1).

**Table 1:** Summary of genetic findings.

**Sample**	**Sequencing method**	**Gene and transcript**	**c.; p**.	**Zygosity**	**ClinVar**	**Mutationtaster prediction**	**gnomAD MAF**	**ACMG classification**	**Age of onset**
1	Trio exome	*NPHS1* NM_004646.4	c.204_207dup; p.(Leu70Alafs*23)	Homo	Not reported	Disease-causing	Not present	Likely pathogenic	3
2	Exome	*NPHS1* NM_004646.4	c.1757G>A; p.(Arg586Lys)	Homo	Not reported	Disease-causing	Not present	VUS	1 month
3	Sanger (after parental exome)	*NPHS1* NM_004646.4	c.2212+2T>A	Homo	Not reported	–	Not present	Likely pathogenic	20 days
4	Exome	*NPHS1* *NM_004646.4*	c.2214T>A; p.(Tyr738*)	Homo	Not reported	Disease-causing	Not present	Likely pathogenic	Congenital
5	Exome	*NPHS1* NM_004646.4	c.3325C>T; p.(Arg1109*)	Homo	Pathogenic	Disease-causing	rs137853042 7.95e-6	Pathogenic	Congenital
6	Exome	*NPHS1* NM_004646.4	c.3250dupG; p.(Val1084Glyfs*12)	Homo	Pathogenic	Disease-causing	rs386833935 7.60e-5	Pathogenic	1 month
7	Exome	*NPHS1* NM_004646.4	c.C3478T:p.(Arg1160*)	Homo	Pathogenic	Disease-causing	rs267606919 9.94e-5	Pathogenic	Congenital
8	Exome	*NPHS1* NM_004646.4	c.3523_3524del; p.(Leu1175Valfs*2)	Homo	Pathogenic	Disease-causing	rs1420307327 3.976e-06	Pathogenic	40 days
9	Trio exome	*NPHS1* NM_004646.4	c.3523_3524del, p.(Leu1175Valfs*2)	Homo	Pathogenic	Disease-causing	rs1420307327 3.976e-06	Pathogenic	4 years
10	Sanger	*NPHS2* NM_014625.4	c.538G>A; p.(Val180Met)	Homo	Pathogenic	Disease-causing	rs74315347 1.196e-05	Pathogenic	3 years
11	Trio exome	*NPHS2* NM_014625.4	c.156del; p.(Thr53Profs*46)	Homo	Pathogenic	Disease-causing	Not present	Pathogenic	10 months
12	Sanger	*NPHS2* NM_014625.4	c.353C>T; p.(Pro118Leu)	Homo	Pathogenic	Disease-causing	rs869025495 Not present	Pathogenic	1.5 years
13	Sanger	*NPHS2* NM_014625.4	c.353C>T; p.(Pro118Leu)	Homo	Pathogenic	Disease-causing	rs869025495 Not present	Pathogenic	1.5 years
14	Sanger	*NPHS2* NM_014625.4	c.467dup; p.(Leu156Phefs*11)	Homo	Pathogenic/Likely pathogenic	Disease-causing	rs530318579 Not present	Pathogenic	NA
15	Sanger	*NPHS2* NM_014625.4	c.G503A; p.(Arg168His)	Homo	Pathogenic	Disease-causing	rs530318579 1.36e-5	Pathogenic	2.5 years
16	Exome	*WT1* NM_024424.6	rs121907910 c.1315C>T; p.(Arg439Cys)	Homo	Pathogenic	Disease-causing	rs121907910 not present	Pathogenic	1 year
17	Exome	*WT1* NM_024424.6	c.1399C>T; p.(Arg467Trp)	Homo	Pathogenic	Disease-causing	rs121907900 Not present	Pathogenic	3 months
18	Exome	*ARHGDIA* *NM_004309.6*	c.275-1G>A; p.(?)	Homo	Not reported	–	Not present	Likely pathogenic	Congenital
19	Trio exome	*NUP205* NM_015135.3	c.3329T>C; p.(Leu1110Pro)	Homo	Likely pathogenic	Disease-causing	rs1584675898 Not present	Pathogenic	1 year
20	Trio exome	*COQ6* *NM_182476.3*	c.782C>T; p.(Pro261Leu)	Homo	Pathogenic	Disease-causing	rs371260604 5.17e-05	Pathogenic	1 year
21	Trio exome	*SGPL1* NM_003901.4	c.1_27del; Start loss	Homo	Not reported	–	Not reported	Likely pathogenic	Congenital
22	Exome	*NPHP1* *NM_001128178.3*	c.386del; p.(Ser129Metfs*53)	Homo	Not reported	Disease-causing	Not reported	Likely pathogenic	2 years
23	Exome	Negative	–	–	–	–	–	–	3.5 months
24	Exome	Negative	–	–	–	–	–	–	6 months
25	Trio exome	Negative	–	–	–	–	–	–	1 year
26	Exome	Negative	–	–	–	–	–	–	7 years
27	Exome	Negative	–	–	–	–	–	–	7 years
28	Trio exome	Negative	–	–	–	–	–	–	Congenital
29	Trio exome	Negative	–	–	–	–	–	–	6 months
30	Trio exome	Negative	–	–	–	–	–	–	4 years

Homo, homozygous; MAF, minor allele frequency; NA, not available; VUS, variant of uncertain significance. Sample 3 was analyzed by Sanger sequencing after parental exome sequencing (ES) due to insufficient DNA quality of the child's sample for ES. Homozygosity of the *NPHS1* c.2212+2T>A variant in the child was subsequently confirmed by Sanger sequencing.

In total, we detected seven variants not previously reported in ClinVar or HGMD, including four different *NPHS1* variants (three variants classified as disease-causing and one VUS) and one novel variant in *ARHGDIA, SGPL1*, and *NPHP1* each (all classified as disease-causing). Amongst the known SRNS disease alleles, we identified *NPHS2* c.353C>T (p.Pro118Leu) as well as *NPHS1* c.3523_3524del (p.Leu1175Valfs^*^2) in two unrelated families each. Three of the four novel *NPHS1* variants as well as variants in *ARHGDIA, SGPL1*, and *NPHP1* all represent novel disease-causing variants as defined by ACMG criteria.

All identified variants except the two variants identified in *WT1* were present in a homozygous state in affected children. Parents were heterozygous carriers of the respective variant. Nine variants represent stop or frameshift variants in *NPHS1, NPHS2*, and *NPHP1* (45%), one allele was a −1 canonical splice site variant in *ARHGDIA*, one a +2 splice site variant in *NPHS1* classified as likely pathogenic according to ACMG criteria, one small deletion causing a start loss in *SGPL1*, and the remaining variants were missense variants. All but 1 variant (*NPHS1* c.1757G>A, p.Arg586Lys, rs730880174; classified as VUS) were classified as disease-causing according to the recommendations of ACMG. Interestingly, rs730880174 in *NPHS1* affecting likewise amino acid 586 but changing it into a glycine instead of a lysine has been reported as pathogenic in ClinVar. Likewise, *NPHP1* c.386del (p.Ser129Metfs^*^53) affects the same amino acid position as rs757139057 (c.385_386del), reported pathogenic in ClinVar.

**Figure 2:**
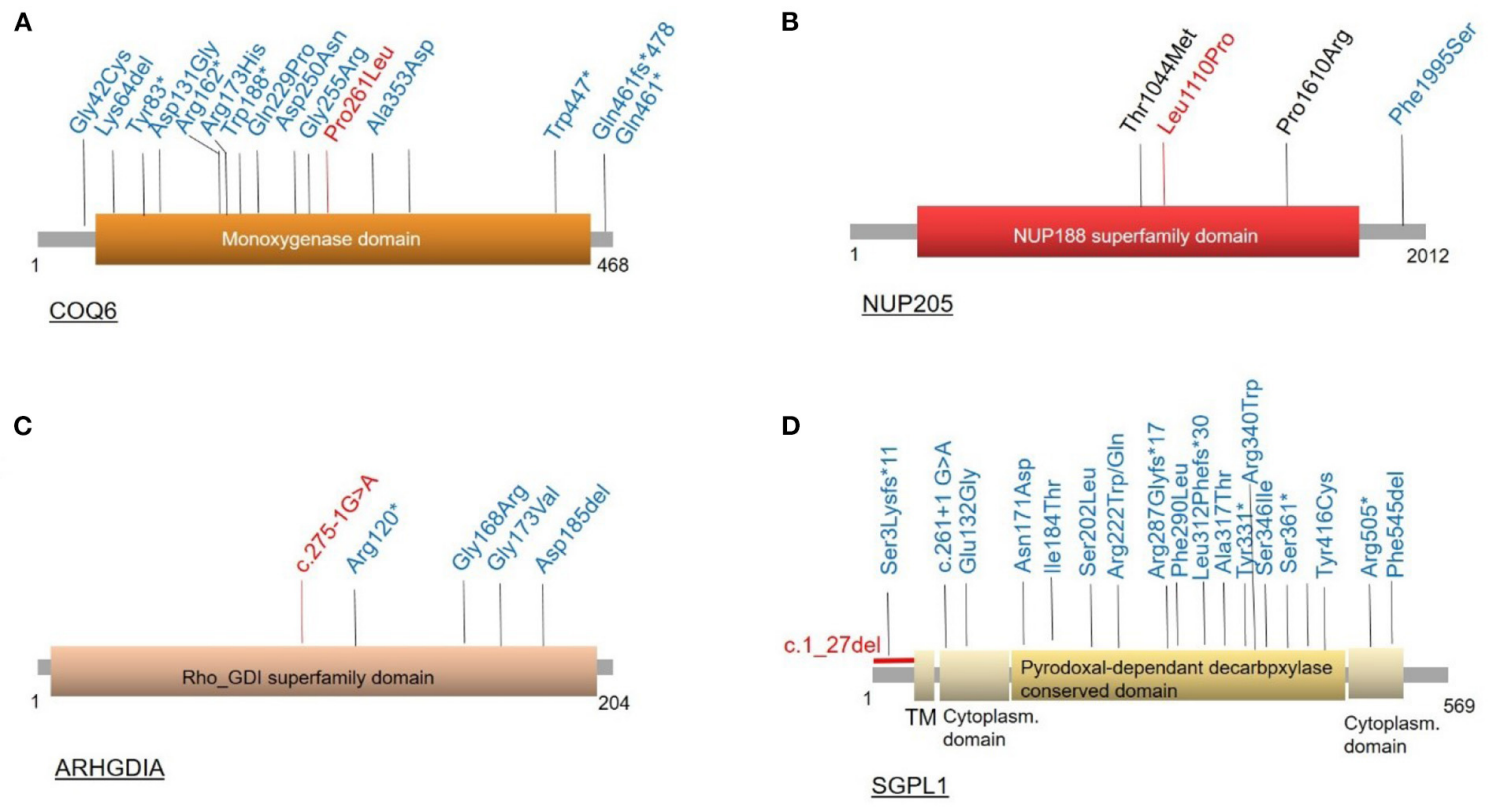
Location of disease-causing variants in **COQ6, NUP205**, **ARHGDIA, **and **SGPL1 **on protein level. **(A)** COQ6, **(B)** NUP205, **(C)** ARHDIA, and **(D)** SGPL1 variants. Previously reported variants are shown in blue, variants first reported in this publication marked in red.

**Figure 3:**
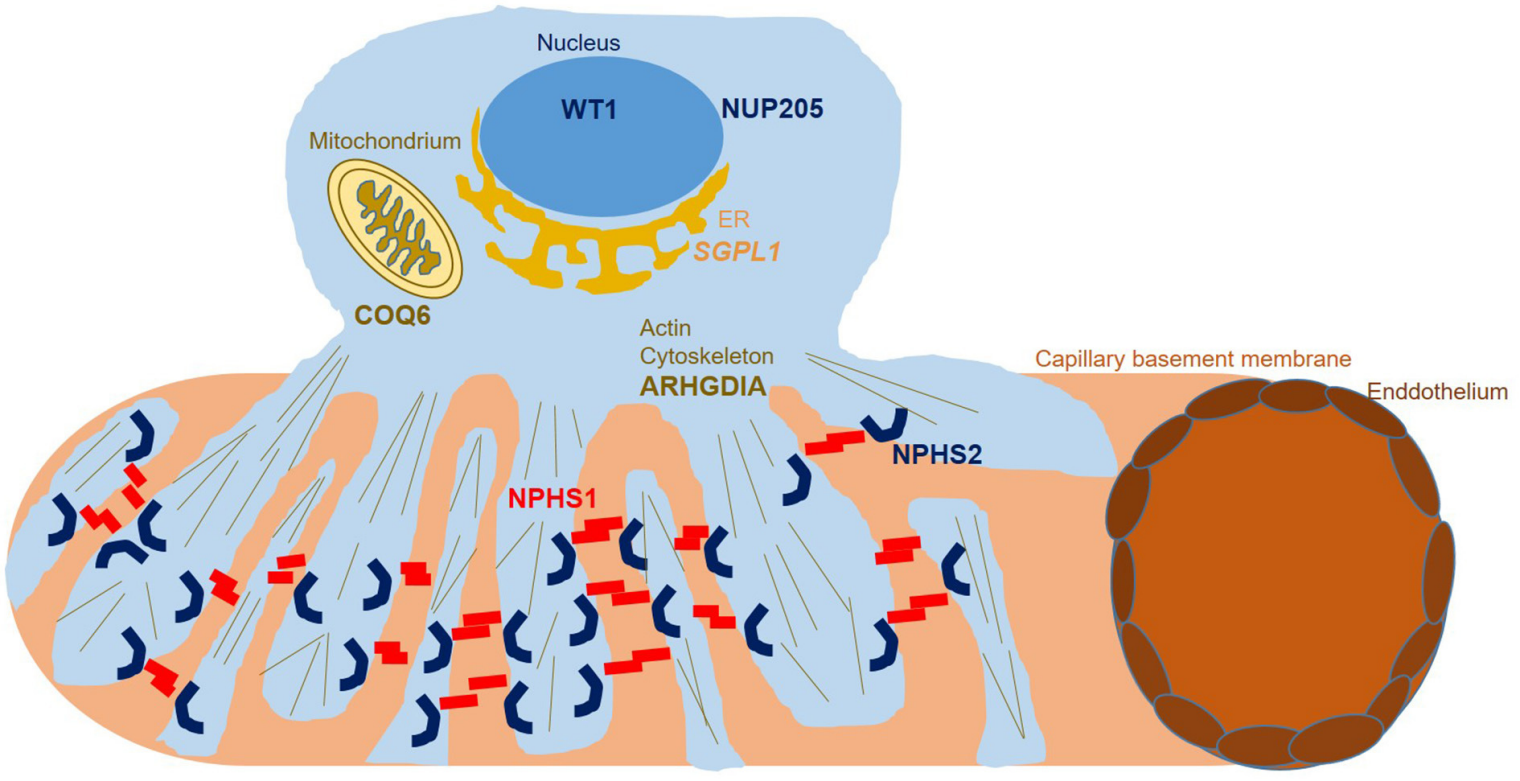
Podocyte subcellular localization of proteins encoded for by genes found to carry disease alleles in this study. While **WT1** functions as a transcription factor in the nucleus, NUP205 functions within the nuclear pore complex, enabling transport between the nucleus and the cytoplasm. **SGPL1** represents an endoplasmic reticulum based enzyme catalyzing sphingolipid breakdown resulting in cleavage of the lipid-signaling molecule sphingosine-1-phosphate. **COQ6** represents a flavin-dependent monooxygenase essential for biosynthesis of coenzyme Q10 which serves as a redox carrier in the mitochondrial respiratory chain as well as an antioxidant protecting cells from reactive oxygen damage. **ARHGDIA** sequesters Rho-GTPases in an inactive state in the cytosol, controlling RhoA, Rac1, and Cdc42 levels and influencing actin dynamics. **NPHS1** and **NPHS2** localize to the podocyte foot process membrane, enabling proper function of the glomerular slit diaphragm.

Both heterozygous missense variants we identified in *WT1* have been reported as pathogenic before with *WT1* c.1315C>T, p.Arg439Cys identified in Denys-Drash syndrome and Meacham syndrome as well as in a Japanese patient with SRNS (15). Variant information for all identified variants and localization of variants in *COQ6, NUP205, ARHGDIA*, and *SGPL1* on protein level are shown in Table 1 and Figures 1, 2. A graphical summary of subcellular localisations and functions of proteins encoded for by genes in which we detected disease-causing variants in this study is shown in Figure 3.

## Discussion

We identified disease-causing variants according to ACMG criteria in genes previously reported to cause childhood SRNS in 21 out of 30 families (70%) and a VUS in one out of 30 families (3%), exceeding the diagnostic yield reported in other larger NGS studies by far (4, 16–18) (Table 2). However, in contrast to outbred study population described in those studies, all of the families referred to us for genetic diagnostics were consanguineous. In addition, it has been previously shown that the diagnostic yield in SRNS is highest in children with congenital nephrotic syndrome and significantly lower with increasing age at first manifestation during the first 6 years of life (18). In line with this, average age at first manifestation in our cohort was 17.5 months and all of our genetically solved cases were below 5 years of age when showing first SRNS symptoms while two of the genetically unsolved cases were 7 years old (Tables 1, 2).

**Table 2:** Comparison of genetic findings in different SRNS cohorts.

**Study**	**Participant number genetically screened**	**Ethnical background**	**Age distribution**	**Diagnostic yield**	***NPHS1* and *NPHS2* diagnostic yield**
Current study	30; *NPHS2* SS + ES	Iran	Median age at onset: 16 months; no patients >7 years of age	Total: 73%; 83% in CNS	*NPHS1*: 30% *NPHS2*: 20%
PodoNet (4)	1,544; 539 screened by NGS panel	85% European, 15% Latin American or Middle East	<20 years	Total: 24%; >60% in CNS	*NPHS1*: 3 % *NPHS2*: 10 %
SRNS study group (18)	1,783; *NPHS2* and *WT1* SS + NGS panel	European, North American, Middle East	<25 years	Total 29.5%; 69.4% in CNS; 49.5% in consang. families	*NPHS1*: 40% <3 months of age; 10% <12 months of age; <5% for older children *NPHS2*: 8.3%
Warejko et al. (17)	335; NGS panel	Mixed	<25 years	Total: 28.7%; 48% in CNS; 38% in consang. families	*NPHS1*: 4.3% *NPHS2*: 2.7%
Wang et al. (19)	110	China	Median age at onset: 4 years	Total: 28.3% 75% in CNS; 68% in familial cases; 24% in sporadic cases	*NPHS1*: 6.4% *NPHS2*: 3.6%
Tan et al. (16)	77	Mixed	<18 years; median age at onset: 3.5 years	Total: 11.1%;	*NPHS1*: 2.7% *NPHS2*: 1.3%
Basiratnia et al. (20)	49; only *NPHS2* screened by SS	Iran	Median age at onset: 7 years	31% for *NPHS2* alone	*NPHS2*: 31%

CNS, congenital nephrotic syndrome; NGS, next-generation sequencing; ES, exome sequencing; SS, sanger sequencing.

Congenital cases are often caused by disease-causing variants in *NPHS1* and we identified such variants in two cases as well as disease-causing variants in *SGPL1* or *ARHGDIA* in one case, respectively. Age of onset was overall lower in *NPHS1* cases compared to *NPHS2* cases, as expected. In total, we most frequently detected biallelic variants in *NPHS1* (38% of solved families and 30% of all families) followed by *NPHS2* (29% of solved families and 20% of all families), in accordance with previous findings that *NPHS1* and *NPHS2* variants are amongst the most common underlying genetic causes in childhood SRNS in non-Iranian populations (17). This is also in line with findings reported by Basiratnia et al. (20) who identified disease-causing variants in *NPHS2* in 15 out of 49 Iranian SRNS cases (31%) and 57% of familiar Iranian SRNS cases. Three of these 15 cases (20%) carried the *NPHPS2* p.Pro118Leu variant homozygously while we detected this variant in 2 of 6 cases (30% of all our *NPHS2* cases), indicating it represents a fairly common Iranian disease allele. We did not detect the previously described Iranian *NPHS2* founder variant p.Arg238Ser reported by Basiratnia et al. (20). Interestingly, the proportion of childhood SRNS cases resulting from biallelic disease-causing *NPHS2* variants seems fairly similar across many populations such as European populations [12–19% in sporadic cases (21, 22), 26% in familiar cases (23)], and 15% in Indian cases (24), while disease-causing *NPHS2* variants are less frequently identified in Chinese, Japanese, or South African probands with SRNS (3–8%) (19, 25–29).

Although we were able to genetically solve 25% of cases by starting with Sanger sequencing of *NPHS2*, in the days of broad availability of NGS, performing NGS panel or ES as primary diagnostic step seems more efficient, given the overall high genetic variability in SRNS. Using ES in *NPHS2* negative cases, we identified multiple biallelic disease-causing *NPHS1* variants, two heterozygous disease-causing *WT1* variants as well as biallelic disease-causing variants in *COQ6, ARHGDIA, NUP205*, and *SGPL*. Disease-causing variants in *COQ6* represent a very rare cause of SRNS with until completion of this study less than 20 disease-causing alleles reported to date in the literature (30, 31) (Figure 2). Very recently, Drovandi et al. reported additional *COQ6* cases as part of a large cohort of 116 individuals with primary coenzyme Q10 deficiency in which CoQ10 replacement had beneficial effects regarding disease progression (32, 33). Disease-causing variants in *ARHGDIA* likewise represent a rare cause of SRNS (Figure 2). ARHGDIA (Rho GDP-Dissociation Inhibitor Alpha) is expressed in podocytes playing an important role in maintaining Rho-GTPases in their inactive state and loss of function subsequently disturbs the actin-cytoskeleton arrangement, resulting in early-onset proteinuria and progressive loss of renal function in humans and mice (34–36). In line with findings in cases previously reported in the literature, our case presented with congenital nephrotic syndrome, confirming a crucial role of ARHGDIA for podocyte integrity. We further identified a homozygous small deletion encompassing exon 2 (the first coding exon) resulting in a start-loss in *SGPL1* in a case with syndromic congenital SRNS. *SGPL1* encodes sphingosine-1-phosphate lyase-1, a ubiquitously expressed enzyme located at the endoplasmic reticulum. Loss of function results in syndromic SRNS and adrenal insufficiency in humans with 20 disease alleles published to date (37, 38) (Figure 2). We also detected a homozygous *NUP205* missense variant, previously reported as disease-causing, in a case with disease onset at 12 months of age. *NUP205* encodes for a nuclear pore complex protein and a different homozygous disease-causing missense variant in *NUP205* has been previously reported in a Turkish sib-pair affected by SRNS (39). Additionally, NUP205 loss of function has been previously associated with left-right patterning defects in humans (40) (Figure 2) as well as ciliogenesis defects in a frog knockdown model system (41). Our case did not exhibit any overt laterality disturbances. A summary of subcellular localisations and functions of proteins encoded for by genes in which variants have been identified in this cohort is shown in Figure 3.

Last, we identified a phenocopy in a case presenting clinically with SRNS where we identified a homozygous disease-causing *NPHP1* frameshift variant. NPHP1 loss of function is a frequent cause of NPHP, with a deletion of *NPHP1* representing the most frequent disease allele (42). Interestingly, Kirsty et al. have previously published a Filipino family with nephrotic syndrome but genetic diagnostics revealing disease-causing variants in *NPHP4* associated with NPHP (43). While proteinuria occurs frequently in NPHP, usually it is not of the nephrotic range and instead of a glomerular protein pattern observed in SRNS, proteinuria in NPHP rather shows a tubular pattern. For our case, unfortunately no clinical reports stating what type of proteins were lost in the urine are available. It therefore remains unclear if the individual suffered from tubular or glomerular proteinuria. We cannot exclude that in addition to the genetically established NPHP, the individual independently also suffered from SRNS of another origin, including a non-monogenic form of SRNS.

Overall, ES was very efficient in our cohort of consanguineous early-onset SRNS cases to establish a genetic diagnosis. Nevertheless, several cases remain unsolved, putatively due to variants outside of the coding regions, warranting genome sequencing (with RNA sequencing) or due to the presence of a polygenic or non-genetic mechanism.

## Data availability statement

The datasets for this article are not publicly available due to concerns regarding participant/patient anonymity. Requests to access the datasets should be directed to the corresponding author.

## Ethics statement

The studies involving human participants were reviewed and approved by Freiburg University ethics commission, votum 122/20 and samples processed under the Radboudumc Diagnostics Innovation programme (CMO2006-048) to establish a genetic diagnosis. Written informed consent to participate in this study was provided by the participants' legal guardian/next of kin.

## Author contributions

MN, AR, EK, SS, TB, AS, AR, PN, SB, MK, and AA recruited the probands and/or were involved in their clinical care. MN, KMR, TM, RB, IS, SS, AK, RB, and MS conducted data analysis. MN and MS conceived the study. MS drafted the manuscript. MN and JH supervised the study. All authors read and approved the final version of the manuscript.

## Funding

IS acknowledges funding from the Freiburg University Medical Faculty Hospital Berta-Ottenstein Clinical fellowship programme. MS acknowledges funding form the European Research Council (ERC): ERC starting grant TREATCilia (grant agreement no. 716344) and MS and AK received funding from the Deutsche Forschungsgemeinschaft (DFG, German Research Foundation)—Project-ID 431984000—SFB 1453 (CRC Nephgen). MS, AK, and RB acknowledge funding from the Deutsche Forschungsgemeinschaft (DFG, German Research Foundation)—Excellence Initiative CIBSS—EXC-2189—Project ID 390939984.

## Conflict of interest

The authors declare that the research was conducted in the absence of any commercial or financial relationships that could be construed as a potential conflict of interest.

## Publisher's note

All claims expressed in this article are solely those of the authors and do not necessarily represent those of their affiliated organizations, or those of the publisher, the editors and the reviewers. Any product that may be evaluated in this article, or claim that may be made by its manufacturer, is not guaranteed or endorsed by the publisher.

## Abbreviations

DNA: deoxyribonucleic acid
ES: exome sequencing
GATK: genome analysis toolkit
LOF: loss of function
OFC: occipitofrontal circumference
PCR: polymerase chain reaction
SD: standard deviation
SNV: single nucleotide variant
SRNS: steroid resistant nephrotic syndrome
SSNS: steroid sensitive nephrotic syndrome.
