# Supplementary material for: High detection rate for disease-causing variants in a cohort of 30 Iranian pediatric steroid resistant nephrotic syndrome cases
Source: Front Pediatr. 2022 Sep 22;10:974840. doi: 10.3389/fped.2022.974840 (PMC9555279; doi:10.3389/fped.2022.974840)
Supplement: Supplementary file 1 [file Table_1.docx]

| **Gene** | **Associated phenotype** | **Inheritance mode** |
| --- | --- | --- |
| [*ACTN4*](https://blueprintgenetics.com/pseudogene/#ACTN4) | Focal segmental glomerulosclerosis | AD |
| *COQ8B* | Nephrotic syndrome | AR |
| *ANLN* | Focal segmental glomerulosclerosis | AD |
| [*APOL1*](https://blueprintgenetics.com/pseudogene/#APOL1) | Sporadic idiopathic steroid-resistant nephrotic syndrome with focal segmental hyalinosis | AD/AR |
| *ARHGAP24* | Familial idiopathic steroid-resistant nephrotic syndrome with focal segmental hyalinosis | AD/AR |
| *ARHGDIA* | Nephrotic syndrome | AR |
| *CD2AP* | Glomerulosclerosis, focal segmental | AR |
| *COL4A3* | Alport syndrome | AD/AR |
| *COL4A4* | Alport syndrome | AD/AR |
| *COL4A5* | Alport syndrome | XL |
| *COQ2* | Coenzyme Q10 deficiency | AR |
| *COQ6* | Coenzyme Q10 deficiency | AR |
| *CRB2* | Focal segmental glomerulosclerosis, Ventriculomegaly with cystic kidney disease | AR |
| [*CUBN*](https://blueprintgenetics.com/pseudogene/#CUBN) | Megaloblastic anemia-1, Finnish | AR |
| *DGKE* | Nephrotic syndrome | AR |
| *DLC1* | Nephrotic syndrome | AR |
| *EMP2* | Nephrotic syndrome | AR |
| *FAN1* | Interstitial nephritis, karyomegalic | AR |
| *FAT1* | Nephrotic syndrome | AR |
| *FN1* | Glomerulopathy with fibronectin deposits 2 | AD |
| *INF2* | Glomerulosclerosis, Charcot-Marie-Tooth disease | AD |
| *ITGA3* | Interstitial lung disease with nephrotic syndrome and epidermolysis bullosa | AR |
| *KANK1* | Cerebral palsy, spastic quadriplegic, 2, Nephrotic syndrome | AD |
| *KANK2* | Palmoplantar keratoderma and woolly hair, Nephrotic syndrome | AR |
| *KANK4* | Nephrotic syndrome | AR |
| *LAGE3* | Galloway-Mowat syndrome 2 | XL |
| *LAMB2* | Nephrotic syndrome, Pierson syndrome | AR |
| *LMX1B* | Nail-patella syndrome | AD |
| *LYZ* | Amyloidosis, systemic nonneuropathic | AD |
| *MAFB* | Multicentric carpotarsal osteolysis | AD |
| *MAGI2* | Nephrotic syndrome 15 | AR |
| *MYO1E* | Focal segmental glomerulosclerosis | AR |
| *NPHS1* | Nephrotic syndrome | AR |
| *NPHS2* | Nephrotic syndrome | AR |
| *NUP107* | Nephrotic syndrome, type 11 | AR |
| *NUP133* | Nephrotic syndrome, type 18 | AR |
| *NUP205* | Nephrotic syndrome, type 13 | AR |
| *NUP85* | Nephrotic syndrome, type 17 | AR |
| *NUP93* | Nephrotic syndrome | AR |
| *OSGEP* | Galloway-Mowat syndrome | AR |
| *PAX2* | Focal segmental glomerulosclerosis 7 | AD |
| *PDSS2* | Coenzyme Q10 deficiency | AR |
| *PLCE1* | Nephrotic syndrome | AR |
| *PTPRO* | Nephrotic syndrome | AR |
| *SCARB2* | Epilepsy, progressive myoclonic | AR |
| *SGPL1* | Nephrotic syndrome 14 | AR |
| *SMARCAL1* | Schimke immunoosseous dysplasia | AR |
| *TBC1D8B* | Nephrotic syndrome, type 20 | XL |
| *TP53RK* | Galloway-Mowat syndrome 4 | AR |
| *TRIM8* | Epileptic encephalopathy | AD |
| *TRPC6* | Focal segmental glomerulosclerosis | AD |
| *TTC21B* | Short-rib thoracic dysplasia, Nephronophthisis, Asphyxiating thoracic dysplasia (ATD; Jeune) | AR |
| *TTR* | Dystransthyretinemic hyperthyroxinemia, Amyloidosis, hereditary, transthyretin-related | AD |
| *WDR4* | Galloway-Mowat syndrome 6 | AR |
| *WDR73* | Galloway-Mowat syndrome | AR |
| *WT1* | Denys-Drash syndrome, Frasier syndrome, Wilms tumor, Nephrotic syndrome, type 4 | AD |
| *XPO5* | Nephrotic syndrome |  |

**Supplemental table 1.** Summary of known SRNS genes prioritized for variant filtering in exome analyses.
